# Learning facial muscle anatomy using virtual reality simulation: a randomized controlled study among medical students in Iraq

**DOI:** 10.1186/s41077-026-00475-2

**Published:** 2026-07-28

**Authors:** Faris Al-Fayyadh, Ali Abdull Sattar Abdull Jabar, Attila Gilányi

**Affiliations:** 1 Doctoral School of Informatics, University of Debrecen, Egyetem tér 1, Debrecen, 4032 Hungary; 2 College of Medicine, Department of Anatomy, University of Sumer, Al-Rifai, Iraq; 3 Faculty of Informatics, University of Debrecen, Egyetem tér 1, Debrecen, 4032 Hungary

**Keywords:** Virtual reality, Simulation-based learning, Anatomy education, Medical education, Facial muscles, Knowledge retention, Perceived enjoyment

## Abstract

**Background:**

Virtual reality (VR) is increasingly used in medical education, particularly for teaching anatomically complex content. However, evidence comparing learning outcomes with conventional screen-based instruction remains limited. This study examined differences in learning outcomes between an immersive, interactive VR-based learning experience and a smart-screen lesson in facial muscle anatomy.

**Methods:**

A randomized controlled trial was conducted with 48 second-year medical students at the University of Sumer, Iraq. Participants completed a 15-item multiple-choice test and were randomly assigned to a VR or smart-screen group. Both groups studied identical content during a 15-minute session. Knowledge was assessed before, immediately after, and two weeks later. Presence and enjoyment were also measured. Data were analysed using repeated measures ANOVA, with additional analyses of learning gain and retention.

**Results:**

Both groups improved after the session. However, the VR condition resulted in significantly higher post-test and follow-up scores. A significant time-by-group interaction indicated different learning trajectories. Additional analyses showed greater learning gains in the VR group, while no significant difference was found in retention. Students in the VR group also reported higher presence and enjoyment.

**Conclusion:**

Immersive and interactive environments may improve short-term learning of complex anatomy. However, retention patterns were similar across conditions, suggesting that differences are mainly due to initial learning rather than sustained retention.

**Supplementary Information:**

The online version contains supplementary material available at https://doi.org/10.1186/s41077-026-00475-2.

## Background

Digital anatomy learning has been explored using different instructional modalities including traditional two-dimensional screen-based methods, three-dimensional visualisations on screens, augmented reality (AR) and fully immersive virtual reality (VR). The degree of immersion and interactivity of these approaches varies. Screen-based methods present learning materials on flat displays, where students mainly observe content with limited interaction. In contrast, immersive VR environments allow learners to actively explore anatomical structures from multiple perspectives, supporting the interpretation of complex spatial regions [1–4]. The growing availability of head-mounted displays has accelerated the adoption of VR alongside augmented and mixed reality technologies, particularly in anatomy and simulation-based training, where safe, repeatable, and engaging learning environments are required [5, 6]. The primary difference among the various delivery mediums for anatomy instruction is the degree of learner interaction with anatomical structures. Traditional educational methods typically allow learners to passively observe and visualize static images of anatomical structures. On the other hand, some interactive multimedia instructional materials have allowed learners to actively manipulate anatomical structures using interactive features including rotation, movement through space (exploration), and changes in viewing perspective.

The way a learner interacts with spatial content influences how information is processed during the learning process. There has been considerable literature comparing learning outcomes from passive versus active presentation styles. Studies comparing immersive and non-immersive media show increased levels of motivation, engagement, and attention in learners using immersive media; however, differences in knowledge outcomes between immersive and traditional approaches are often modest [7–9]. Meta-analytic studies suggest that VR can improve knowledge to a moderate extent and often leads to higher learner satisfaction [10]. However, the findings are not fully consistent. Some studies report better performance with VR, while others show that immersive VR and conventional three-dimensional applications can produce similar outcomes [11, 12]. These mixed findings suggest that learning outcomes are shaped not only by the technology itself, but also by how the learning activity is designed [13, 14]. In anatomy education, these considerations are particularly relevant for spatially complex regions. Studies in craniofacial and head-and-neck anatomy show that immersive and three-dimensional approaches can support the interpretation of layered structures and spatial relationships more effectively than traditional methods [15–17]. However, most existing work focuses on surgical or specialised training contexts rather than undergraduate education. Additional studies show that learning in VR often develops through active interaction with the material during the activity itself [18, 19].

Immersive VR in this study is defined as a head-mounted display–based environment where users can move around, rotate, and explore three-dimensional anatomical models. The term “smart-screen” refers to traditional screen-based teaching, where conventional screen displays (e.g., PowerPoint presentation on a two-dimensional display) are mainly viewed by learners without directly interacting with anatomical structures. Presence in the context of VR is the subjective feeling and sensation of “being there”, and it indicates how much a learner feels immersed and engaged in the virtual environment. In this study, presence was measured as perceived presence using questionnaire items.

Despite the increasing use of VR in anatomy education, the application of immersive learning to facial muscle anatomy remains largely unexplored. Facial muscles form a dense and overlapping system. Understanding them requires a clear three-dimensional view of structure, orientation, and how the parts relate to each other. This makes them difficult to study using conventional screen-based approaches. VR has been used in areas such as craniofacial and aesthetic anatomy. However, no previous study has directly examined its effectiveness for undergraduate learning of facial muscles. Given these distinctions, there remains a need to examine how an immersive and interactive learning experience compares with a conventional screen-based approach, particularly in relation to knowledge acquisition and short-term retention. This gap forms the basis of the present study, which compares an immersive, interactive VR-based learning experience with a conventional smart-screen lesson in facial muscle anatomy. The study aims to examine differences in learning outcomes, knowledge retention, and learning experience across the two instructional conditions.

### Research questions

In line with the objectives of this study and the identified research gap, the following research questions were formulated.

- RQ1. Does learning anatomy using VR lead to greater learning gains compared to smart-screen learning?
- RQ2. Is knowledge retention after two weeks higher for students who learned using VR compared to those who used a smart screen?
- RQ3. Do students report different levels of presence and perceived enjoyment between VR and smart-screen learning methods?
- RQ4. How do students describe their learning experience when using VR compared to smart-screen learning?

## Methods

### Study design and ethical approval

This study used a randomized controlled design. It compared a VR session with a smart-screen lesson. The study was conducted between October and December 2025 at the College of Medicine, University of Sumer, Iraq. Participation was voluntary, and all students gave informed consent before taking part. Ethical approval was obtained from the Research Ethics Committee of the College of Medicine, University of Sumer (Approval No. 14, Date: 15-10-2025).

### Participants and allocation

Second-year medical students enrolled in anatomy courses were invited to participate. All students had prior exposure to facial anatomy. None had previous experience with VR-based learning. A total of 52 students agreed to participate. Participants were assigned to two groups using a simple randomization method. A random number generator was used to create the allocation sequence prior to the sessions. The allocation sequence was prepared in advance and was not accessible to participants prior to group assignment.

Participants were assigned to either the VR group or the smart-screen group according to the pre-generated allocation sequence by the research team prior to the sessions. Four participants withdrew during the study. In total, forty-eight participants were included (twenty-three in the Virtual Reality [VR] condition; twenty-five in the Smart Screen condition). Participants were aged between 19 and 21 years. Both male and female students were included. An identification code was assigned to each participant for use across all data collection procedures. All participants were enrolled in the same course and had comparable academic backgrounds, and none had prior experience with virtual reality-based learning. Therefore, baseline leftacteristics between groups were similar. Blinding was not feasible due to the nature of the intervention. However, anonymized participant codes were used during data collection and analysis to reduce potential bias.

### Learning conditions

#### VR condition

**Fig. 1:**
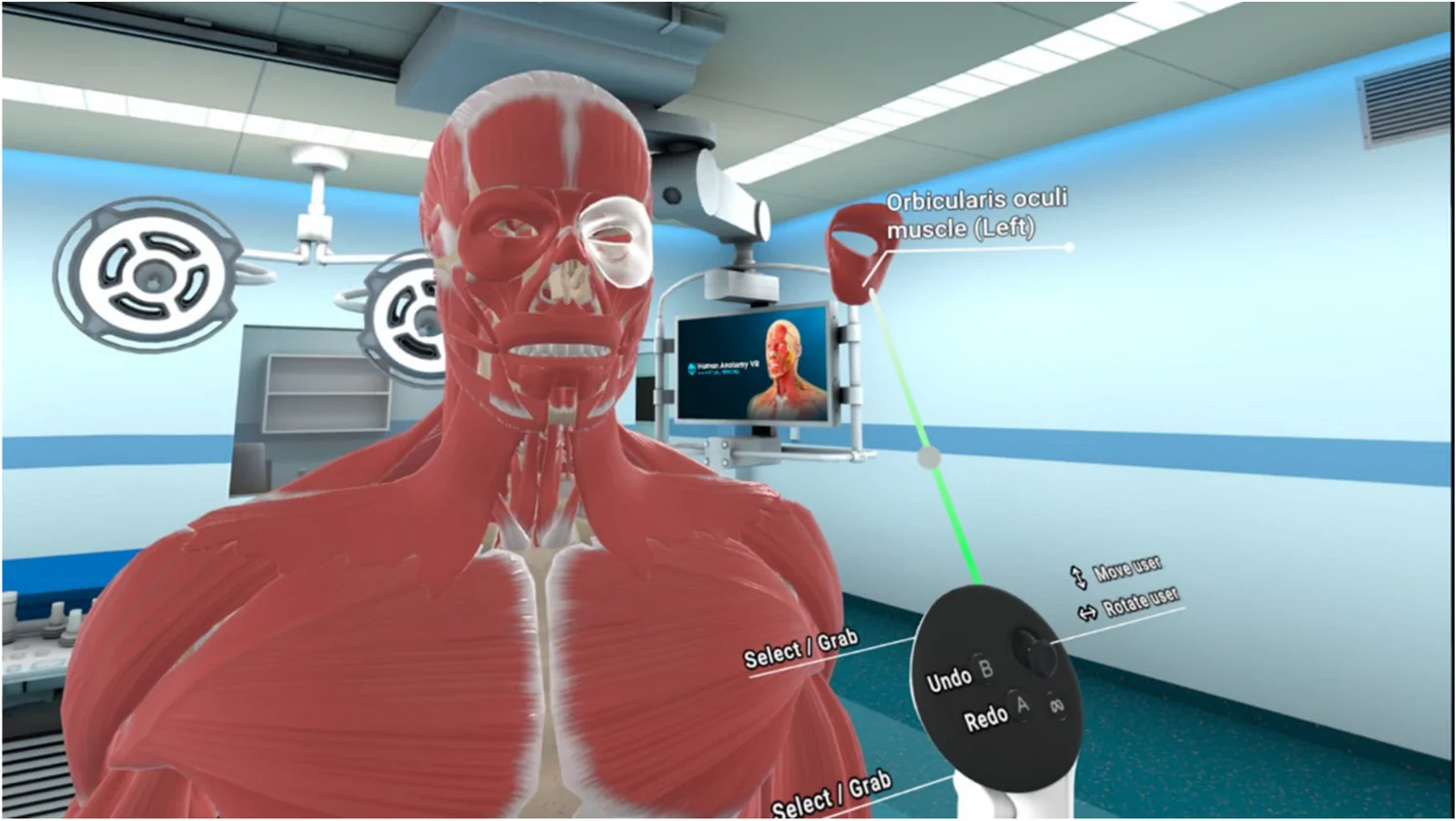
Interactive three-dimensional anatomy model used in the VR condition, illustrating how students selected and examined facial muscles (e.g., orbicularis oculi) using a handheld controller during the learning session

Students in the VR group completed a 15-minute session using an Oculus Quest 3 headset. The 15-minute duration was selected to provide equivalent exposure across both instructional conditions while allowing participants an opportunity to explore the anatomical content. The learning activity focused on a specific anatomical region (facial muscles) rather than a complete anatomy module, and therefore a brief but focused session was considered sufficient for content exploration. A relatively brief session was also considered appropriate to minimise potential discomfort during first-time VR use [20]. The session began with a brief orientation phase. Students were shown how to use the headset and its basic controls. The objective of the session was to support students’ understanding of the structure, identification, and spatial relationships of facial muscles. Following the orientation phase, students used the *Human Anatomy* VR application to explore an interactive three-dimensional model of facial muscles. The model was interactive. Students could rotate it, zoom in, and select individual muscles to see their names and details. The session was designed to support active exploration of spatial relationships between structures. Students worked individually. The session was supervised by an anatomy instructor who was also the course lecturer for facial anatomy. The orientation phase was included within the 15-minute session. Students were not allowed to ask content-related questions during the session, and no external observers were present. The instructor was present to provide basic guidance if needed, but no additional teaching was provided during the session (see Fig. 1).

#### Smart-screen condition

Students in the smart-screen group attended a 15-minute PowerPoint lesson. The content was the same as in the VR condition and was delivered by the same instructor. The format was two-dimensional and non-interactive. Students only observed the material without interacting with the anatomical structures. Both conditions were matched in content, duration, and instructor. The instructor provided minimal guidance in both conditions and did not add explanations beyond the planned session content. The two conditions differed in delivery, interactivity, and engagement. The comparison reflects an immersive and interactive experience versus a conventional screen-based format. All VR sessions were completed individually using separate devices under controlled conditions.

### Outcome measures

A knowledge test was developed for use in this study. Test items were designed to reflect key learning objectives related to facial muscle anatomy. Two experienced anatomy instructors reviewed the test to ensure alignment with the learning objectives, content accuracy, and clarity of the items. Minor revisions were made based on their feedback. This process provided evidence of content validity; however, the test was not formally pilot-tested or psychometrically validated (e.g., no reliability analysis was conducted) prior to the study. The full set of test items can be found in Appendix A.

The test was designed to measure students’ ability to understand concepts related to anatomy as opposed to simply recalling names or locations. The test was administered at three time points: before the session, after the session, and two weeks later. For each of these administrations, the same 15 multiple-choice questions were administered to each participant. The order of the questions and the order of the possible answers for each item were randomized to minimize familiarity effects. All of the test administrations were conducted under identical conditions. Each participant’s response to each question on the test was scored based on the number of correct answers. Participants also completed a 5-item questionnaire assessing their perceived presence. Perceived enjoyment was measured using three items. Responses were collected using a five-point Likert scale. The presence items were informed by established presence measures [21]. The enjoyment items were informed by established motivation measures [22]. Internal consistency was acceptable. Cronbach’s alpha was 0.823 for presence and 0.807 for enjoyment. Students also responded to four open-ended questions after the session. These questions focused on their learning experience, perceived benefits, and challenges.

### Procedure

After randomization, students attended their assigned session. The VR group completed the session using the headset. The smart-screen group attended the PowerPoint lesson. All tests and questionnaires were completed using participant codes. The timing of all assessments was identical for both groups.

### Statistical analysis

**Fig. 2:**
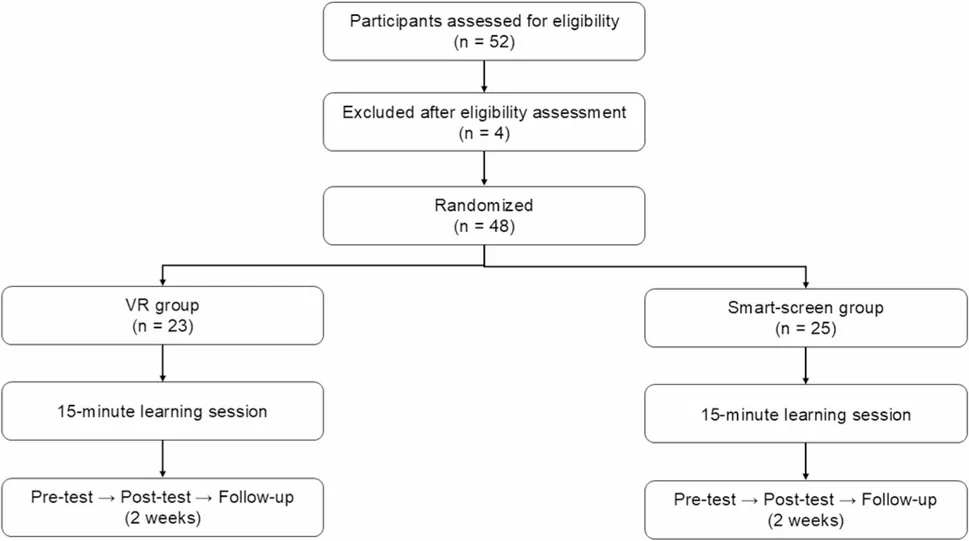
Study design and participant flow for the randomized controlled trial

Data were analysed using SPSS version 27. Normality was tested using the Shapiro–Wilk test. Homogeneity of variance was examined using Levene’s test. Learning outcomes were analysed using repeated measures ANOVA. Time (pre-test, post-test, follow-up) was treated as the within-subject factor, and group (VR vs. smart-screen) as the between-subject factor. Descriptive statistics (means and standard deviations) were calculated. The significance level was set at 0.05. A formal a priori power analysis was not conducted, as the sample included the full cohort of students enrolled in the course. Open-ended responses were analysed descriptively to provide illustrative insights into students’ experiences, rather than through a formal qualitative analysis approach. These responses were used to complement the quantitative findings. The study design and participant flow of the randomized controlled trial are presented in Fig. 2.

## Results

### Descriptive statistics

Students’ performance was assessed at three time points: before the learning session, immediately after the session, and two weeks later. At the pre-test stage, scores were low in both groups and showed little difference. The VR group recorded a mean score of 4.46, while the smart-screen group scored 4.40. After the learning session, scores increased in both conditions. In the post-test, students in the VR group achieved higher scores (M = 9.46) than those in the smart-screen group (M = 8.20). At the follow-up stage, scores declined in both groups. The VR group maintained higher mean scores (M = 7.00) compared to the smart-screen group (M = 5.88). Table 1 presents the descriptive statistics for test scores across the three measurement points for both learning conditions.

**Table 1:** Descriptive statistics of test scores across the three measurement points

Measurement point	VR group (Mean ± SD)	Smart-screen group (Mean ± SD)
Pre-test	4.46 ± 1.62	4.40 ± 1.66
Post-test	9.46 ± 1.74	8.20 ± 1.71
Follow-up	7.00 ± 1.84	5.88 ± 1.54

### Assumption checks

The assumptions required for repeated measures ANOVA were examined. Mauchly’s test of sphericity was not significant (*p* = .865), indicating that the sphericity assumption was met. Therefore, results assuming sphericity were used. Levene’s test showed no significant differences in error variances between groups at any measurement point (all *p* > .05), confirming that the assumption of homogeneity of variances was satisfied.

### Effects of time (within-subjects effect)

A significant main effect of time was found, F(2, 94) = 158.96, *p* < .001, partial η² = 0.772, showing changes in performance across the three measurement points.

### Effects of group (between-subjects effect)

A significant main effect of group was observed, F(1, 47) = 4.38, *p* = .042, partial η² = 0.085. When scores were averaged across all measurement points, the VR group showed higher performance than the smart-screen group.

### Interaction between time and group

**Fig. 3:**
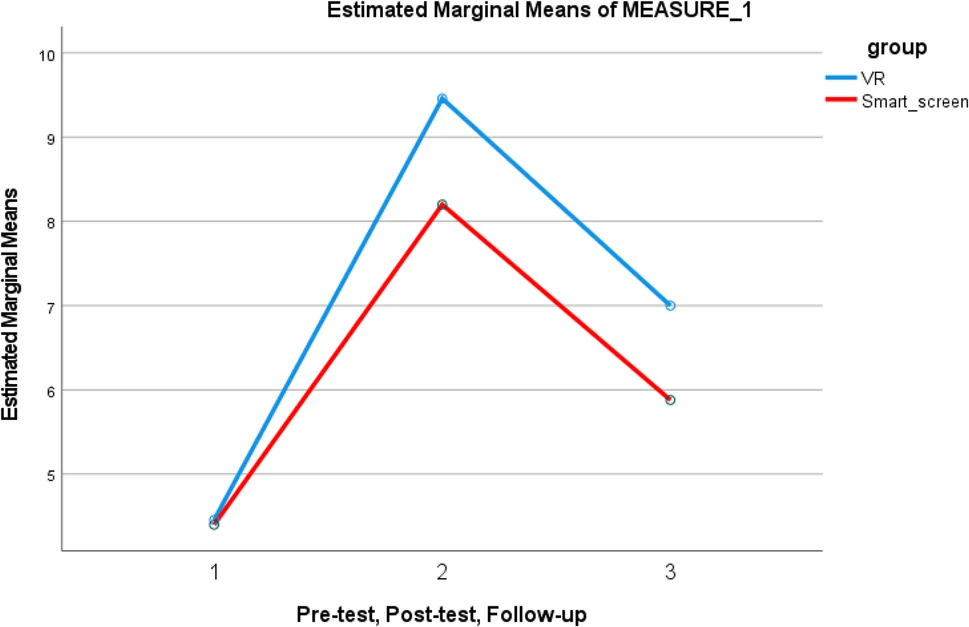
Mean test scores for the VR and smart-screen groups across pre-test, post-test, and follow-up

The interaction between time and group was significant, F(2, 94) = 3.53, *p* = .033, partial η² = 0.070. This result shows that scores changed differently over time in the two groups. As shown in Fig. 3, both groups started with similar pre-test scores. Scores increased in both groups after the session, with a greater increase in the VR group. At the follow-up stage, scores decreased in both groups, with the VR group maintaining higher absolute scores, although retention patterns did not differ significantly between conditions.

At the post-test stage, the VR group scored significantly higher than the smart-screen group (*p* = .014, 95% CI [0.26, 2.25]). A similar between-group difference was also observed at the follow-up stage, where the VR group maintained significantly higher scores than the smart-screen group (*p* = .025, 95% CI [0.15, 2.09]), supporting the significant time-by-group interaction.

To further examine differences in learning gain, gain scores (post-test minus pre-test) were calculated and compared between groups. An independent-samples t-test showed that the VR group achieved significantly greater learning gains (M = 5.00, SD = 1.38) than the smart-screen group (M = 3.80, SD = 2.06), t(47) = 2.38, *p* = .021, with a moderate effect size (Cohen’s d = 0.68).

To examine retention patterns, retention scores (follow-up minus post-test) were calculated and compared between groups. An independent-samples t-test showed no significant difference between the VR group (M = -2.46, SD = 1.67) and the smart-screen group (M = -2.32, SD = 1.65), t(47) = -0.29, *p* = .772, indicating a similar decline in performance over time across both conditions.

### Post-hoc comparisons

Pairwise comparisons confirmed significant differences between all-time points (all *p* < .001). Scores increased significantly from pre-test to post-test, followed by a significant decline at the follow-up stage. Follow-up scores remained significantly higher than pre-test scores.

### Presence questionnaire results

An independent-samples t-test was conducted to compare perceived presence between the VR and smart-screen groups. Students in the VR condition reported significantly higher levels of presence (M = 3.78, SD = 0.70) than those in the smart-screen condition (M = 3.38, SD = 0.61), *t*(47) = 2.13, *p* = .039. The effect size was moderate (Cohen’s *d* = 0.61). The five-item presence scale demonstrated good internal consistency (Cronbach’s α = 0.823), indicating acceptable reliability [23].

### Perceived enjoyment results

Students in the VR group reported higher levels of perceived enjoyment (M = 4.04, SD = 0.65) than those in the smart-screen group (M = 3.61, SD = 0.59). An independent-samples t-test showed that this difference was statistically significant, t(47) = 2.41, *p* = .020. The effect size was medium (Cohen’s d = 0.69). The three-item perceived enjoyment scale showed good internal consistency (Cronbach’s α = 0.807) [23].

### Open-ended responses

Across both learning conditions, students’ statements about test results or their own performance were rare. Instead, they generally discussed what occurred during the session, focusing on what happened at each stage of the learning process. Students also tended to describe short moments and specific actions during the session more frequently than the overall activity. However, differences did emerge in how students described their interaction with the material. Stability and ease of use were emphasised. References to interaction with the content were less frequent and, when mentioned, were typically described as limited. Students typically stated that learning occurred by watching or following the content. Students typically stated that learning occurred by simply watching or following the content. The VR experience showed a different pattern. In VR, students mentioned interacting with the anatomical model. Others focused on moving around the structures to see how they were arranged. These comments suggest that students were actively exploring the content, rather than just observing it.

At the start of the VR sessions, some students mentioned difficulties with the controls. Others noted brief technical issues or physical discomfort, such as eye strain or the weight of the headset. These comments often appeared alongside positive remarks about the experience. In several cases, students described both ease of engagement and moments of discomfort within the same session. Some also reflected on whether they would want to use this method again. As they did so, a few expressed a clear willingness to repeat the experience. Others were more reserved, suggesting that their choice would depend mainly on practical factors, such as comfort with the technology and the length of the session.

## Discussion

This study compared immersive VR with a traditional smart-screen lesson for learning facial muscle anatomy. Clear differences were found between the two learning methods, both in learning outcomes and in perceived presence.

### Learning outcomes over time

Short-term learning was observed in both groups when examining test results across the different time points. Both formats appeared to support immediate learning for all participants. Differences between the two groups became more noticeable over time. An important point is that additional analyses indicated that there was no significant difference in retention between the two groups. That being said, the differences observed at the follow-up stage appear to be mainly due to greater initial learning gains in the VR group rather than better retention over time. These patterns can be understood in relation to how the learning activities were experienced. This difference may be related to how students processed the content during the session. In the VR condition, students were not only viewing the material, but also interacting with it by changing perspective and focusing on specific structures. This type of interaction may support how learners organise spatial information, as understanding develops through direct engagement with the content rather than passive observation. In contrast, the smart-screen condition presented the same material in a more observational format, with limited opportunities for interaction.

This may help explain the higher performance observed across measurement points in the VR condition. This is particularly relevant for topics such as facial muscle anatomy, where understanding depends on recognising how anatomical structures are arranged and related to each other. Similar patterns have been reported in anatomy education, particularly in topics involving complex spatial structures [24]. However, it is important to note that the present comparison reflects differences in both delivery format and level of interaction, rather than VR as an isolated factor. Therefore, the observed differences should not be attributed to VR alone. Factors such as interactivity, learner engagement, and the novelty of the experience may have contributed to the outcomes observed in this study.

### Group differences and learning retention

Although results indicate that students in the VR condition performed better than those in the smart-screen condition, this comparison should be interpreted with caution, as the two conditions differed not only in delivery format but also in the level of interactivity and learner engagement. In addition, the interaction between time and instructional method suggests that learning developed differently across the two conditions. Specifically, scores in the VR group increased across time points, whereas scores in the smart-screen group showed a clearer decline at the follow-up stage. Moreover, additional analyses indicated that retention patterns did not differ significantly between the two groups. Therefore, the observed differences over time appear to be mainly related to how information was initially acquired rather than how it was maintained. The inclusion of the two-week follow-up test was aimed at investigating whether the learning gains observed immediately after the intervention persisted beyond the learning session. Even though the intervention was of short duration, a delayed test provides a clearer indication of any learning that may have occurred compared to a post-test carried out immediately after the intervention. According to previous studies on education, it is important to conduct delayed tests when measuring learning outcomes as opposed to just measuring immediate learning outcomes [25]. Also, delayed tests have been conducted in virtual reality studies to measure persistence of learning outcomes [20]. Thus, a two-week follow-up assessment was included to examine whether any learning benefits remained evident after the initial learning experience.

The intervention was intentionally limited to 15 min. The primary aim of the study was to compare two instructional approaches under equivalent learning conditions rather than to examine the effect of training duration. Therefore, all participants received the same content and the same exposure time. A 15-minute session allowed learners to explore and interact with the anatomical structures while maintaining a practical and manageable learning activity. In addition, previous evidence suggests that longer exposure to immersive VR may increase the likelihood of cybersickness and user discomfort [26]. Despite the intervention being only 15 min long, the students did not just look at the anatomical information. In the VR session, they examined the facial muscles from various angles and interacted directly with the three-dimensional model. Understanding facial anatomy depends largely on recognising how structures are arranged and related to one another, and VR provided an opportunity to examine these relationships in a more direct way [27]. For this reason, it was reasonable to examine whether some of the learning gained during the session remained evident beyond the immediate post-test.

The way learners interacted with the material may also help explain the observed differences. Students who used virtual reality (VR) had the opportunity to view multiple aspects of the anatomy directly and manipulate them as needed. Viewing the material in this way may have helped them understand how the different parts of a structure fit together, especially in topics involving layered and closely related structures such as facial muscles. Students in the smart-screen group primarily viewed images and had limited opportunities to manipulate the material. Overall, the results suggest that differences in learning outcomes may depend on the level of interaction learners have with the subject matter. In the VR condition, students engaged in active manipulation of anatomical structures, which required continuous spatial processing and interpretation of relationships between elements. This form of interaction is consistent with models of immersive and interactive learning, where learners construct mental representations through direct engagement with content [27, 28]. The main reason for the different results appears to be the nature of the smart-screen and VR conditions, particularly the degree of learner involvement and control over the presentation of the material. In contrast to attributing the results to VR technology alone, the findings point to interactivity and experiential engagement as key contributors to learning [29]. It should be noted that the knowledge assessment focused on understanding spatial relationships rather than simple recall, which aligns with the cognitive demands of the VR activity, where learners were required to explore, interpret, and organise anatomical structures during the session. In this sense, the assessment captures learning outcomes that are aligned with how the content was processed, strengthening the link between the learning experience and the observed performance.

### Presence and learning experience

The descriptions of the two learning activities by participants differed. During VR experiences, students typically reported on what they were doing during the session rather than focusing on outcomes or scores. A greater sense of involvement was noted when students could actively manipulate anatomical models. Conversely, when interaction was reduced and observation became more dominant, this sense of involvement was less evident. Tasks that required spatial relationships appeared to involve different forms of attention during the learning activity. In anatomy, understanding often depends on recognising where structures are located and how they relate to each other. Being able to approach the material from different angles may have supported how students engaged with this information, particularly when the visual content was complex or layered [1].

Previous studies report similar observations, although not always in the same form. For example [30], found that students learning mediastinal anatomy in VR showed higher interest and clearer spatial understanding, even when differences in test performance were limited. VR-based activities have also been associated with spatial reasoning [14]. Other studies report that immersive environments are used to support the understanding of spatial relationships, even when overall performance remains similar across conditions [31]. In these cases, learners often describe how they engage with the material during the activity rather than focusing on final outcomes [7].

The main differences in how students approached the learning process were related to the technology used. The smart-screen condition was often described as familiar and easy to manage. Comfort and ease of use were emphasised, while interaction with the material was mentioned less frequently. In contrast, many students in the VR condition described a more active experience. They referred to examining structures in three-dimensional space, moving around the models, and adjusting their viewpoint to understand how elements were arranged. These observations indicate that students described different forms of engagement across the two conditions. In addition, students in the VR condition reported higher perceived enjoyment during the session. Their descriptions often included active exploration of the learning material. Some students also reported initial physical or technical difficulties, such as eye strain or discomfort with the headset or controls. These issues were mainly mentioned at the beginning of the session and became less prominent as students became more familiar with the system.

### Student reflections

This section addresses Research Question Four (RQ4) by describing how students reflected on their learning experience when using VR compared to smart-screen learning. Many students described their learning experiences in terms of what they were doing during the session rather than in relation to scores or final outcomes. Their responses remained close to specific actions and moments that occurred while interacting with the material. When comparing both conditions, students in the VR condition focused more on how they engaged with the visual content. They described moving around the anatomical models, adjusting their viewpoint, and returning to specific structures as needed. These actions were often described alongside maintaining attention during the session. Students described their understanding in relation to this interaction. Many students indicated that learning developed gradually through exploration rather than as a single outcome measured at the end of the session.

In contrast, students in the smart-screen condition described a different pattern. They described the session as familiar and easy to manage. Comfort and stability were emphasised, but interaction with the content was more limited. Learning was more often described as observational, with fewer opportunities for active involvement during the session. Students rarely referred to correctness or test performance in either condition. Rather than focusing on what they learned, students described how they engaged with the material. The smart-screen condition was often described as easy and predictable, while the virtual reality (VR) condition was more often linked to exploration and active involvement. These observations reflect differences in how students described their learning experience across the two conditions.

### Educational implications

The research shows there isn’t a single type of task for students to complete when it comes to learning; many types of tasks have different requirements from learners (particularly within subjects like anatomy, which requires spatial understanding). A number of tasks can rely upon more traditional approaches to format (i.e., recognising ‘basic’ information), while tasks requiring the learner to understand how structures relate spatially appear to require learners to engage with these differently. The learning experience in the Smart-Screen condition was viewed by students as being comfortable and easy to navigate. The Smart-Screen condition is likely best suited for applications that have either time constraints; resource limitations; or a lack of consistent access to technological assistance. The Smart-Screen condition is well-suited for providing students with a consistent and predictable presentation of organised and structured information. Where student interaction is not an essential aspect of the instructional activity, the Smart-Screen condition has many advantages.

VR condition provided students with the ability to engage with the same course content differently than they did in the Smart-Screen condition. While students were interacting with similar content, their interaction in the VR condition involved movement; exploring objects within the virtual world; and adjusting perspective to view objects from a variety of angles. Students’ actions as they interacted with objects also influenced how they processed the course content during the session. As such, interaction in the VR condition was not merely another tool for instructors to provide supplemental information; instead, it was an integral component of the learning process. These findings indicate that VR is likely to have the greatest potential when the content has to be explored actively (as opposed to passively) and organised in space.

The contribution of VR in this research therefore lies primarily in the way that it enables students to interact with their subject matter in ways which support both the development of their spatial understanding and the organisation of their knowledge. On the other hand, consideration needs to be given to practical issues when using VR in an educational context. For example, initial adjustment to the system, physical comfort, and ease of use may influence how students experience a particular activity, especially at the beginning of their first exposure. As such, a short period of orientation and thoughtful session design are important for effective implementation. VR may also be introduced into anatomy education through the inclusion of small amounts of VR within typical lesson timeframes. There are several reasons for this; first, students need an opportunity to become familiar with the technology prior to using it during activities. Second, it is beneficial to keep these sessions as short as possible to minimise student discomfort while they adapt to the virtual environment. Third, allowing students the flexibility to work at their own pace and revisit difficult areas or structures when needed appears to support students’ ability to manage spatial information. Finally, instructors benefit from providing clear and concise guidance rather than overwhelming students. These recommendations are based on observations from this study. However, because the design elements examined were not tested independently, these findings should be interpreted with caution.

### Limitations and future work

This study was conducted at a single institution; therefore, all participants were drawn from the same cohort of second-year medical students. Although the sample size was limited, it included the full group of students enrolled in the course, which reduces selection bias but means that the findings reflect one specific institutional context. Therefore, the results should not be generalised to other higher education settings. The follow-up assessment was conducted approximately two weeks after the learning session. This allowed for the observation of short-term retention; however, it does not provide information about how learning may develop over longer periods or after repeated exposure. It remains unclear whether the observed patterns would persist over time or change as students become more familiar with the learning format.

An important limitation of this study is that it did not isolate VR as a single variable. The differences between the two conditions were not limited to the delivery method (VR vs. screen), but also included the level of interactivity and learner engagement. As a result, the findings reflect an immersive and interactive learning experience rather than the effect of VR alone. The physical setting of the VR sessions may also have influenced the results. All VR activities were conducted in standard laboratory spaces rather than in environments designed specifically for immersive use. Movement was restricted, and comfort was not always optimal. At the same time, these conditions reflect the reality of many educational settings where dedicated VR facilities are not always available. Therefore, the observed differences should be interpreted as reflecting a combination of immersion, interactivity, and initial engagement rather than a pure effect of VR. In addition, the knowledge test was not formally pilot-tested or psychometrically validated prior to the study, which may limit the strength of the measurement and the interpretability of the findings. Although the order of questions and answer options was varied across measurement points to reduce familiarity effects, some degree of familiarity with the test items may still have influenced performance.

Participants entered the study with no prior experience with immersive VR. As a result, responses may have been influenced by the novelty of the technology during initial exposure. Some participants reported discomfort or difficulty using the system during the early stages of the session; however, these issues became less prominent as the session progressed, suggesting that adjustment occurred through use. Another limitation relates to the short duration of the intervention. The study was based on a single learning session; therefore, the findings reflect immediate and short-term responses rather than long-term educational outcomes. It remains unclear how learning and engagement would develop over extended use or repeated exposure. As familiarity with the technology increases, the influence of novelty may change over time.

Future research should examine how these findings develop over longer periods and across different learning contexts. It would also be useful to separate the effects of immersion, interactivity, and instructional design to better understand how each factor contributes to learning outcomes. In addition, studies involving multiple institutions and larger samples would help to improve the generalisability of the findings.

## Conclusion

Both learning conditions led to improved knowledge after the session. However, the VR condition resulted in higher performance at both the post-test and follow-up stages, reflecting greater initial learning gains. Additional analyses indicated that retention patterns did not differ significantly between groups, suggesting that the observed differences were primarily related to how information was acquired rather than how it was maintained over time. These findings highlight the value of immersive and interactive learning environments in supporting the understanding of complex spatial anatomy. Rather than replacing conventional approaches, VR may be most effective when used alongside existing instructional methods to enhance active engagement with learning content.

## Supplementary Information

Supplementary Material 1.

## Data Availability

The datasets used and/or analysed during the current study are available from the corresponding author on reasonable request.

## Abbreviations

VR: Virtual reality
AR: Augmented reality
MR: Mixed Reality
HMD: Head-mounted display
CBCT: Cone-beam computed tomography
ANOVA: Analysis of variance
