# Supplementary material for: Learning facial muscle anatomy using virtual reality simulation: a randomized controlled study among medical students in Iraq
Source: Adv Simul (Lond). 2026 Jul 28;11:79. doi: 10.1186/s41077-026-00475-2 (PMC13693077; doi:10.1186/s41077-026-00475-2)
Supplement: Supplementary file 1 [file 41077_2026_475_MOESM1_ESM.docx]

**Appendix A**

Knowledge Test – Facial Muscle Anatomy

Instructions: Choose the correct answer for each question. Select the single best answer.

**1. Which nerve supplies most of the muscles of facial expression?**
A) Trigeminal nerve (CN V)
B) Facial nerve (CN VII)
C) Glossopharyngeal nerve (CN IX)
D) Vagus nerve (CN X)

**2. The frontalis muscle is primarily responsible for:**
A) Closing the eyelids
B) Raising the eyebrows
C) Compressing the cheeks
D) Pursing the lips

**3. Which muscle encircles the eye and closes the eyelids tightly?**
A) Orbicularis oris
B) Orbicularis oculi
C) Buccinator
D) Zygomaticus major

**4. Damage to the facial nerve at the stylomastoid foramen results in:**
A) Loss of sensation over the face
B) Paralysis of facial expression muscles
C) Difficulty in chewing
D) Loss of taste from the posterior third of the tongue

**5. Which muscle helps in smiling by elevating the corner of the mouth?**
A) Zygomaticus major
B) Platysma
C) Buccinator
D) Depressor anguli oris

**6. The zygomaticus minor muscle mainly helps to:**

A) Retract the lower lip
B) Elevate the upper lip
C) Depress the angle of the mouth
D) Compress the cheeks

**7. Which of the following muscles compresses the cheeks during blowing or whistling?**
A) Buccinator
B) Zygomaticus minor
C) Levator labii superioris
D) Mentalis

**8. The orbicularis oris muscle functions to:**
A) Open the mouth widely
B) Close and protrude the lips
C) Elevate the upper lip
D) Retract the corners of the mouth

**9. Which branch of the facial nerve supplies the orbicularis oculi muscle?**
A) Temporal branch
B) Buccal branch
C) Marginal mandibular branch
D) Cervical branch

**10. The muscle that wrinkles the nose is:**
A) Nasalis
B) Levator anguli oris
C) Zygomaticus minor
D) Mentalis

**11. Which muscle depresses the lower lip?**
A) Depressor labii inferioris
B) Depressor anguli oris
C) Mentalis
D) Platysma

**12. The mentalis muscle acts to:**
A) Elevate the upper lip
B) Raise and protrude the lower lip
C) Retract the angle of the mouth
D) Compress the cheeks

**13. The levator labii superioris muscle acts to:**

A) Elevate the upper lip
B) Lower the lower lip
C) Retract the lips laterally
D) Close the mouth

**14. Which muscle contributes to the “frowning” expression?**

A) Corrugator supercilii

B) Zygomaticus major

C) Frontalis

D) Levator labii superioris

**15. The depressor anguli oris muscle acts to:**

A) Elevate the corner of the mouth
B) Depress the corner of the mouth
C) Raise the upper lip
D) Compress the cheeks

**Appendix B**

Student Perception and Experience Questionnaire

**Part I. Likert-Scale Items**

| Item | Strongly disagree | Disagree | Neutral | Agree | Strongly agree |
| --- | --- | --- | --- | --- | --- |
| 1- **Presence:** |  |  |  |  |  |
| I felt like I was present inside the learning environment. |  |  |  |  |  |
| The learning environment felt realistic and immersive. |  |  |  |  |  |
| I felt surrounded by the learning content. |  |  |  |  |  |
| I had a strong sense of being inside the learning environment. |  |  |  |  |  |
| I was able to focus on the learning content without being distracted by the surrounding environment. |  |  |  |  |  |
| 2- **Perceived Enjoyment:** |  |  |  |  |  |
| I find using this learning method enjoyable. |  |  |  |  |  |
| Using this learning method is pleasant. |  |  |  |  |  |
| I have fun using this learning method. |  |  |  |  |  |

**Part II. Open-Ended Questions**

1. What did you think about the learning experience using this method, and why?
2. Would you like to use this learning method again in the future? Please explain your answer.
3. Which aspects of this learning method influenced your understanding of the content? Please describe specific features (e.g., clarity, focus, comfort, interaction, navigation).

4. Did this learning method change how you understood or visualised the content? Please explain.
